# 

**Published:** 1919-08-30

**Authors:** 


### The Book Market.

**List of New Books Received from the following publishers:—**

H. K. Lewis and Co., Ltd.: "Notes on Galvanism and Faradism." By E. M. Magill, M.B., B.Sc. 6s. net. "Modern Medicine and Some Modern Remedies." By T. B. Scott, M.R.C.S., L.R.C.P. (Second Edition.) 6s. 6d. net.

Longmans, Green and Co.: "Shell-Shock and its Lessons." By Professor G. Elliot Smith, M.D., F.R.S., and T. H. Pear, M.A., B.Sc. 1s. 6d. net.

The Albany Press (Fitzroy Square, W. 1): "Lectures on Venereal Diseases." By Dr. Leonard Myers. 6s.

Cassell and Co.: "The Welfare of the Expectant Mother." By Mary Scharlieb, C.B.E., M.D., M.S. 5s. net.

Sampson Low, Marston and Co., Ltd.: "The Natural History of the Child." By Dr. Courtney Dunn. 7s. 6d. net.

Wm. Heinemann, Ltd.: "The Practitioner's Manual of Venereal Diseases." By A. C. Magian, M.D. 10s. 6d. net.

### OLD PIPES.

Many hospitals which received wounded soldiers will remember the useful supply of pipes which came through the organisation in connection with the officers and staff of the Metropolitan Railway Co., who collected and renovated many thousand old pipes for the use of the wounded. An interesting little booklet containing illustrations has been issued describing the subject by Mr. James Feiron, whose idea and initiative commenced the organisation.

### Gifts and Bequests.

Mr. J. L. Gibbons, J.P., of Sedgley, Staffs, M.P. for South Wolverhampton, 1898-1900, left £2,000 to the Wolverhampton Hospital for Women.

"B. R. F." has given £1,000 to the Royal Hospital and Home for Incurables, Putney, to name in perpetuity a bed to the memory of his sister.

Mr. James Hugh Allen, a shipowner, of Worcester, has bequeathed £10,000 each to the Worcester Infirmary and the Liverpool Cathedral Fund.

Mr. Jacob Usher, J.P., of Trowbridge, has left £500 each to the Trowbridge Cottage Hospital and the Children's Home, Bonnor Road, N.

Mr. W. L. Gladstone, of Liverpool, has bequeathed £1,000 to the Liverpool Royal Infirmary, £700 to the Royal Southern Hospital, £500 each to the David Lewis Northern Hospital, Stanley Hospital, Blue Coat Hospital, School for the Indigent Blind, and Seamen's Orphanage, and £2,000 to such other charities as the executors and the Mayor of Liverpool may select.

Mrs. Sarah Hewitt, of Ipswich, left £250 each to the St. John Ambulance Association, Ipswich Centre, the Ipswich Institution for the Blind, and the Mental Hospital, Colchester. The residue of her estate was bequeathed to the East Suffolk and Ipswich Hospital.

Huddersfield Royal Infirmary has received £2,000 from Mr and Mrs. William Shires, Huddersfield, for the endowment of cots; £1,000 from Mr. and Mrs. E. J. Bruce, Huddersfield, for the endowment of cots; and £1,000 from Mr. W. Pilling, Marsden, for the endowment of a bed.

De Beers Consolidated Mines, Ltd., has made a donation of £500 each to the Hospital for Women, Soho Square, the Chelsea Hospital for Women, the Royal Waterloo Hospital for Women and Children, Queen Charlotte's Hospital, the Elizabeth Garrett Anderson Hospital, the Royal National Pension Fund for Nurses, Mount Vernon Hospital, and the National Hospital for the Paralysed and Epileptic.

